# Long-Read Single Molecule Sequencing to Resolve Tandem Gene Copies: The *Mst77Y* Region on the *Drosophila melanogaster* Y Chromosome

**DOI:** 10.1534/g3.115.017277

**Published:** 2015-04-09

**Authors:** Flavia J. Krsticevic, Carlos G. Schrago, A. Bernardo Carvalho

**Affiliations:** * Centro Internacional Franco Argentino de Ciencias de la Información y de Sistemas, CONICET, Ocampo y Esmeralda, S2000EZP Rosario, Argentina; † Departamento de Genética, Universidade Federal do Rio de Janeiro, 21941-971, Rio de Janeiro, Brazil

**Keywords:** Y chromosome, *Drosophila melanogaster*, long-read assembly, PacBio, *Mst77F*

## Abstract

The autosomal gene *Mst77F* of *Drosophila melanogaster* is essential for male fertility. In 2010, Krsticevic *et al.* (*Genetics* **184:** 295−307) found 18 Y-linked copies of *Mst77F* (“*Mst77Y*”), which collectively account for 20% of the functional *Mst77F*-like mRNA. The *Mst77Y* genes were severely misassembled in the then-available genome assembly and were identified by cloning and sequencing polymerase chain reaction products. The genomic structure of the *Mst77Y* region and the possible existence of additional copies remained unknown. The recent publication of two long-read assemblies of *D. melanogaster* prompted us to reinvestigate this challenging region of the Y chromosome. We found that the Illumina Synthetic Long Reads assembly failed in the *Mst77Y* region, most likely because of its tandem duplication structure. The PacBio MHAP assembly of the *Mst77Y* region seems to be very accurate, as revealed by comparisons with the previously found *Mst77Y* genes, a bacterial artificial chromosome sequence, and Illumina reads of the same strain. We found that the *Mst77Y* region spans 96 kb and originated from a 3.4-kb transposition from chromosome 3L to the Y chromosome, followed by tandem duplications inside the Y chromosome and invasion of transposable elements, which account for 48% of its length. Twelve of the 18 *Mst77Y* genes found in 2010 were confirmed in the PacBio assembly, the remaining six being polymerase chain reaction−induced artifacts. There are several identical copies of some *Mst77Y* genes, coincidentally bringing the total copy number to 18. Besides providing a detailed picture of the *Mst77Y* region, our results highlight the utility of PacBio technology in assembling difficult genomic regions such as tandemly repeated genes.

The *Mst77F* gene of *Drosophila melanogaster* is essential for male fertility and encodes a component of sperm chromatin (Raja and Renkawitz-Pohl 2005; Barckmann *et al.* 2013). Russell and Kaiser (1993) found three Y-linked pseudogenes of this gene (*Mst77*-ψ1, *Mst77*-ψ2, and *Mst77*-ψ3) by sequencing testis complementary DNAs. Years later the genome sequence of *D. melanogaster* became available (Adams *et al.* 2000), but unfortunately it was not very informative on the Y-linked paralogs of *Mst77F* (“*Mst77Y*” hereafter), in part because of the general difficulties of sequencing and annotation of Y-linked genes (Carvalho *et al.* 2009), and mostly because these genes were severely misassembled (Krsticevic *et al.* 2010). For example, several *Mst77Y* genes (most with incomplete sequences) could be found in the then-available genome assembly (and until Release 5), but none of them corresponded to the pseudogenes described by Russell and Kaiser (Krsticevic *et al.* 2010). Given the misassemblies, Krsticevic *et al.* (2010) used a *de novo* sequencing approach, polymerase chain reaction (PCR) of *Mst77Y*-like genes followed by cloning and sequencing, and found 18 *Mst77Y* genes. Because the DNA came from an inbred strain (the same used in genome sequencing), each different gene sequence variant was inferred to correspond to a different gene. To circumvent PCR-induced artifacts Krsticevic *et al.* (2010) sequenced more than 100 clones and only picked gene sequence variants occurring twice or more. They also used two other methods that yielded similar estimates of the number of *Mst77Y* genes: restriction enzyme digestion plus band quantification (16 copies) and computational analysis of the whole-genome sequencing traces (14 copies). Ten *Mst77Y* genes (named as *Mst77Y-3*ψ, *Mst77Y-5*ψ, etc.) have disrupted open reading frames (with premature stop codons and indels), and hence are pseudogenes; some of them seem to correspond to the previously described *Mst77*-ψ1 and *Mst77*-ψ2 (Russell and Kaiser 1993). Interestingly, the remaining eight *Mst77Y* genes (*Mst77Y-1*, *Mst77Y-2*, *Mst77Y-4*, *Mst77Y-7*, *Mst77Y-8*, *Mst77Y-9*, *Mst77Y-12*, and *Mst77Y-13*) have intact open reading frames, and several of them are expressed and correctly spliced in testis, accounting for ∼20% of the functional *Mst77F*-like mRNA. Finally, purifying selection was detected, mostly in the potentially functional copies. Hence, some *Mst77Y* genes are functional genes (Krsticevic *et al.* 2010).

Using Y-chromosome deletions, Russell and Kaiser (1993) broadly mapped the *Mst77Y* genes to h18-hl9 heterochromatic bands, whereas Krsticevic *et al.* (2010) showed that the duplicated region (or at least what survived from it) spans ∼3.4 kb of the 3L chromosome, encompassing the whole *Mst77F* gene, and pieces of two neighboring genes (two exons of *Pka-R1*, and the 5′-UTR of *CG3618*). The current *D. melanogaster* genome assembly (Release 6; Hoskins *et al.* 2015) incorporated the data from Krsticevic *et al.* (2010) and, hence, the 18 *Mst77Y* genes appear there, although with several mismatches. However, the genomic structure and context of the *Mst77Y* region, its length on the Y chromosome, and the possible existence of additional copies of *Mst77Y* genes remained unknown. Long-read technologies, which are being developed in the last few years (Clarke *et al.* 2009; Eid *et al.* 2009; Voskoboynik *et al.* 2013), are specially suited for resolving repetitive regions because repeats only create assembly problems when their length exceeds the read length, *i.e.*, as read length grows the chance of collapsing paralogous copies drops. For example, if each Y-translocated 3.4-kb copy of chromosome 3L is flanked by a small amount of single-copy DNA, an assembly with read length greater than ∼4 kb should not collapse different copies (as happened with the Sanger-based assembly) even if they are 100% identical. The recent availability of two long-read assemblies of *D. melanogaster* (PacBio: Berlin *et al.* 2014; Kim *et al.* 2014; Illumina Synthetic Long-Reads: McCoy *et al.* 2014) prompted us to reinvestigate this challenging region of the Y chromosome.

## Materials and Methods

### *D. melanogaster* assemblies

#### Illumina Synthetic Long-Reads assembly:

As detailed in McCoy *et al.* (2014), DNA from mixed males and females of the reference genomic strain ISO1 (*y*; *cn*, *bw*, *sp*; the same used in the *Drosophila* Genome Project) was sequenced by use of the Illumina TruSeq Synthetic Long-Reads technology and assembled with the Celera Assembler. This assembly (“SLR”) was downloaded from NCBI (accession JAQD00000000.1).

#### PacBio assemblies:

DNA from adult males of the ISO1strain of *D. melanogaster* was sequenced with PacBio technology (Kim *et al.* 2014); the reads were assembled with the Celera Assembler using the recently developed MHAP algorithm as the overlapper (Berlin *et al.* 2014). This assembly (“MHAP”) was downloaded from NCBI (accession JSAE00000000.1). We also examined two preliminary assemblies of the same reads: PBcR used the standard PBcR pipeline (instead of MHAP) and the Celera Assembler (http://cbcb.umd.edu/software/pbcr/dmel.html), whereas FALCON used PacBio’s in house FALCON assembler (http://blog.pacificbiosciences.com/2014/01/data-release-preliminary-de-novo.html). These assemblies were downloaded from http://cbcb.umd.edu/software/pbcr/dmel_cons_asm.tar.gz and http://datasets.pacb.com.s3.amazonaws.com/2014/Drosophila/reads/dmel_FALCON_diploid_assembly.tgz. The Celera Assembler usually produces a main assembly and another set of scaffolds, called “degenerate,” that contains less-reliable sequences (Hoskins *et al.* 2002). Because we have found before that degenerate scaffolds contain pieces of Y-linked genes (Krsticevic *et al.* 2010) we used the two sets of scaffolds while searching for the *Mst77Y* genes in the MHAP and PBcR assemblies. PacBio reads, used to check the assemblies, were downloaded from http://gembox.cbcb.umd.edu/mhap/raw/dmel_filtered.fastq.gz.

#### Sanger assembly:

As a comparison reference for the aforementioned long-read assemblies, we used the WGS3 assembly, which is the best unfinished Sanger assembly of the *D. melanogaster* genome (Hoskins *et al.* 2002). We have not used Release 6 because it incorporated the data from Krsticevic *et al.* (2010) (Hoskins *et al.* 2015) and hence cannot be used as an independent reference. WGS3 was assembled with the Celera assembler and hence include a set of degenerate scaffolds (called “armUextra”). It was downloaded from ftp://ftp.fruitfly.org/pub/download/compressed/WGS3_het_genomic_dmel_RELEASE3-0.FASTA.gz.

### Annotation of the *Mst77Y* region in the long-reads assemblies

All BLAST searches were run locally in a Linux server. The sequences of the 18 *Mst77Y* genes (Krsticevic *et al.* 2010) were used in BLASTN searches for the identification and annotation of scaffolds containing the *Mst77Y* genes in the different long-read assemblies. Note that the sequences of *Mst77Y* genes deposited in 2010 (GQ868243−GQ868260) correspond to their coding sequences and hence omit their small intron. Different *Mst77Y* genes may be 99.5% identical, and to better identify them, we used here the gene sequence (*i.e.*, with the intron; accessions KP684500−KP684517) instead of the coding sequences. We also annotated the misassembled regions (*Misassembly detection using Illumina reads* section) and transposable elements (with *RepeatMasker*; Smit *et al.* 1996–2010). We then inspected the *Mst77Y* region, searching for missing and new *Mst77Y* genes, misassemblies, other genes, *etc*., with all information displayed with the *IGV* browser (Thorvaldsdottir *et al.* 2013).

### Misassembly detection using Illumina reads

Casey Bergman *et al.* generated a large dataset of 100 bp paired-end Illumina from ISO1 adult males and kindly made it available at http://bergman.smith.man.ac.uk/data/genomes/2057_Illumina.tgz . We used it for misassembly detection in the long-read assemblies, employing two different approaches. First, we inspected in the *IGV* browser the *bwa*-generated alignment (Li and Durbin 2009), searching for regions of zero coverage (*i.e.*, where Illumina reads failed to align) or consistent mismatches. Zero-coverage regions also were detected and quantified using the *bedtools* suite of programs (Quinlan and Hall 2010). Second, we used the *YGS* program, which decomposes both the assembled genome and the Illumina reads in *k*-mers and compare the two lists, searching the genome for *k*-mers that are not matched by the Illumina-derived *k*-mers (Carvalho and Clark 2013). Given the high coverage of the Casey Bergman’s Illumina data (∼90x for the autosomes; ∼45× for the sex-chromosomes), its source (males from the same ISO1 strain used in the assembly), and the inherently low error rate of Illumina sequencing, genomic *k*-mers that are unmatched by the Illumina reads almost certainly are due to assembly errors (or to new mutations in the ISO1 strain). We displayed the location of the unmatched *k*-mers in *IGV* as well. The two approaches of misassembly detection are complementary, as they do not always flag the same regions (Supporting Information, Figure S1). The parameters and scripts used with the *bwa*, *bedtools*, *YGS*, and other programs are available with the authors upon request.

## Results and Discussion

### Synthetic Long Reads assembly

Table 1 summarizes the analysis of *Mst77Y* genes in the two main long read assemblies (SLR and MHAP), as well as in two preliminary PacBio assemblies (PBcR and FALCON), and in the Sanger WGS3 assembly. The SLR assembly recovered more *Mst77Y* sequences than the Sanger-based WGS3 assembly and most of them are error-free, but still is incomplete and fragmented. The scaffolds are small (all less than 15 kb), and hence provide little information on the genomic structure and context of the *Mst77Y* region. These problems most likely are a direct consequence of this technology. Synthetic long reads are made by sequencing bar-coded ∼10 kb genomic fragments with standard Illumina short-reads, performing a local assembly of these 10-kb fragments into synthetic long reads, and then feeding them into a standard assembler (Voskoboynik *et al.* 2013; McCoy *et al.* 2014). The method works well with repetitive DNA as long as there is only one copy of a repeat in each 10-kb fragment, *i.e.*, the repeats should be interspersed. Indeed, SLR has been shown to perform well in reconstructing transposable elements (McCoy *et al.* 2014), which are the prototypical interspersed repeat. Tandem repeats, however, are expected to be misassembled and, as we found with the PacBio assembly, this is precisely the case of the *Mst77Y* region: 10-kb genomic fragments frequently will contain two or three highly similar *Mst77Y* genes. It is worth noting also that several biologically interesting and poorly known regions of the *Drosophila* genome, such as other recently duplicated genes, the histone and rDNA clusters, and the centromeres, have a tandem repeat organization, and in these cases synthetic long reads are predicted to have limited utility.

**Table 1:** *Mst77Y* genes in different assemblies of the *D. melanogaster* genome

Assembly	*Mst77Y* Genes Found	Perfect Matches*^a^*	With Errors	Number of Scaffolds	Scaffold Size, kb
SLR	10	8	2	7	3−13
MHAP	18	18	−	1	747
PBcR	20	17	3	2	20; 177
FALCON	18	11	7	1	619
WGS3	6	2	4	6	<2

a 100% identical over the entire length to some gene described in Krsticevic *et al.* (2010).

### MHAP assembly and description of the *Mst77Y* region

The MHAP assembly recovered 18 *Mst77Y* genes in a single contig (accession JSAE01000257; Table 1), without any mismatch with the sequences described by Krsticevic *et al.* (2010). Given this, and also the absence of misassembly signs in the intergenic regions [*Search for misassemblies in the Mst77Y region (PacBio assemblies)* section], we conclude that MHAP is at least a fairly accurate reconstruction of the *Mst77Y* region, and used it to analyze this region (the two preliminary PacBio assemblies will be commented in a next section).

**Figure 1:**
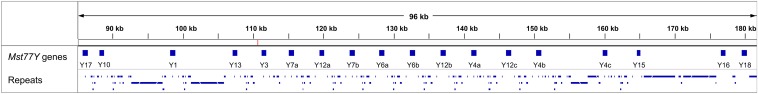
General view of the *Mst77Y* region (MHAP assembly). All 18 *Mst77Y* genes are located in a single contig (JSAE01000257). Gene names were abridged (*Mst77Y-1* as “Y1,” *Mst77Y-17*ψ as “Y17,” and so forth). All genes have the same orientation (not visible at this scale). The red tick near 110 kb marks the unmatched *k*-mer found in this region (caused by a C/T substitution in an intergenic region). The pseudogenes of *Pka-R1* and *CG3618*, which flank each *Mst77Y* gene, were omitted for the sake of clarity. Repeats (mostly retrotransposons) occupy 48% of the sequence.

**Figure 2:**
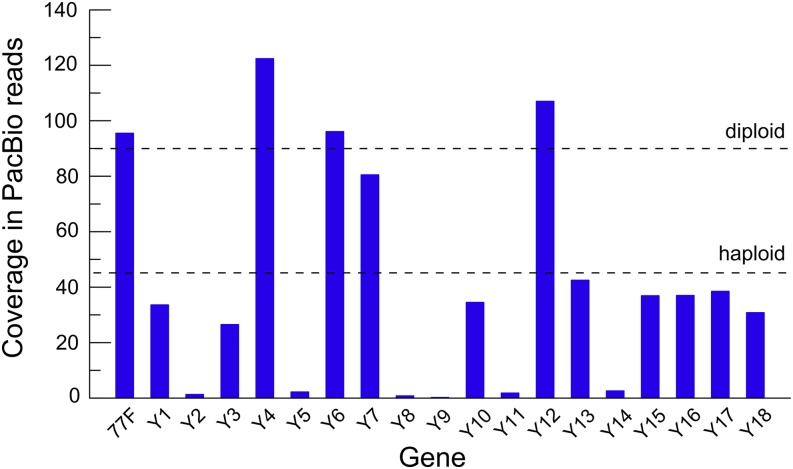
Validation of *Mst77Y* genes by alignment with PacBio reads. Gene names were abridged: *Mst77F* to *77F*, *Mst77Y-1* to *Y1*, and so forth. PacBio reads were aligned with *bwa* against the 18 *Mst77Y* genes identified by Krsticevic *et al.* (2010), and alignment depth was calculated with *bedtools*. Sequencing depth is ∼90× for autosomes (dashed line marked with “diploid”) and ∼45× for sex-chromosomes (“haploid” dashed line; http://bergmanlab.smith.man.ac.uk/?p=2176). Note that the six genes absent from the assembly (Y2, Y5, Y8, Y9, Y11, and Y14) have essentially zero coverage, and hence are artifacts (see *MHAP assembly and description of the Mst77Y region* section). Note also that Y6 and Y7 behave as diploids (and indeed have two copies in the assembled scaffold), whereas the coverage of Y4 and Y12 suggest three copies (which indeed are found in the assembly). We used PacBio reads before “polishment” (*i.e.*, error correction), so these are essentially raw reads.

As summarized in Figure 1, the *Mst77Y* genes are located in tandem over 96 kb, with the same orientation. Some genes are present in identical multiple copies: *Mst77Y-4* and *Mst77Y-12* have three copies, whereas *Mst77Y-6*ψ and *Mst77Y-7* have two copies. As Krsticevic *et al.* (2010) noted, the “gene sequence variant counting” method they used could not detect identical copies, so their discovery is somewhat expected. On the other hand, we could not find six genes described in Krsticevic *et al.* (2010): *Mst77Y-2*, *Mst77Y-5*ψ, *Mst77Y-8*, *Mst77Y-9*, *Mst77Y-11*ψ, and *Mst77Y-14*ψ. These missing genes may be due a misassembly in MHAP or to an experimental artifact in Krsticevic *et al.* (2010). Two lines of evidence strongly suggest that the second hypothesis is true. First, these six genes also are missing in the other assemblies listed in Table 1. Second, supposing that they were misassembled in MHAP, they must be present in the PacBio reads, because of their high coverage of the genome (∼90× for the autosomes, 45× for the sex-chromosomes). Therefore, we aligned with *bwa* these raw reads to the 18 *Mst77Y* genes described by Krsticevic *et al.* (2010), plus the autosomal *Mst77F*, and measured the coverage of each gene. The result (Figure 2) is a stunning confirmation of the findings reported above: the six missing genes are absent from the reads (their coverage is essentially zero). Furthermore, the multiple copy *Mst77Y* genes have a much greater coverage, similar to the autosomal (hence, diploid) *Mst77F*, whereas the remaining *Mst77Y* genes have the lower coverage expected for single-copy Y-linked genes (hence, haploid). We have not carried an analogous test using Illumina reads because they are too short to be unambiguously mapped to each *Mst77Y* gene.

It is worth discussing the origin of these six artifactual genes. Krsticevic *et al.* (2010) sequenced 115 clones of PCR products of the *Mst77Y* genes. To guard against PCR-induced errors, which would mimic additional *Mst77Y* genes, the authors did two separate PCR-cloning experiments (one with *Taq* and one with a *Pfu* low-error polymerase) and only considered gene sequence variants occurring at least twice. It turns out that this is insufficient: if PCR-induced “mutations” occur in early cycles they can attain rather high frequencies, and this is the likely explanation for the these six genes (see also Schenk *et al.* 2006). Interestingly, the six genes do not contain any new substitution (as would be expected from point mutations); rather, they all seem to originate from recombination among the other *Mst77Y* genes. For example, the *Mst77Y-8* sequence can be generated by pasting the 5′ of *Mst77Y-4* with the 3′ of *Mst77Y-16*ψ, whereas the opposite combination (5′ of *Mst77Y-16*ψ with the 3′ of *Mst77Y-4*) would generate *Mst77Y-14*ψ. It has been suggested that *Pfu* generates more recombination artifacts than *Taq* (Zylstra *et al.* 1998), and indeed all six artifactual *Mst77Y* genes came from the PCR-cloning experiment that used this polymerase [see Table 1 of (Krsticevic *et al.* 2010)]. We suggest that readers planning this type of experiment should avoid *Pfu*, and strictly follow the recommendations of Schenk *et al.* (2006).

Besides the *Mst77Y* genes, the 96-kb region contains pieces of two flanking genes (the first two exons of *Pka-R1*, and the 5′-UTR of *CG3618*; *Mst77F* is located inside an intron of *Pka-R1*), in the same order which they occur in 3L, and also many transposable elements (mostly retroelements), which account for 48% of the region.

We should note that it was just a coincidence that the number of *Mst77Y* genes (18 genes) remained the same in Krsticevic *et al.* (2010) and in the present paper: six genes were discarded as PCR artifacts (*Mst77Y-2*, *Mst77Y-5*ψ, *Mst77Y-8*, *Mst77Y-9*, *Mst77Y-11*ψ, and *Mst77Y-14*ψ), and six other genes were added as newly detected duplicates (*Mst77Y-4b*, *Mst77Y-4c*, *Mst77Y-12b*, *Mst77Y-12c*, *Mst77Y-6*bψ, and *Mst77Y-7b*). We followed *Drosophila*’s standard nomenclature and named the duplicated genes as *Mst77Y-4a*, *Mst77Y-4b*, *Mst77Y-4c*, and so forth.

### Search for misassemblies in the *Mst77Y* region (PacBio assemblies)

Given its repeat-rich composition and the lack of a reference sequence for most of its length (the exception being the *Mst77Y* genes), it would be desirable to have some independent validation of the MHAP assembly of the *Mst77Y* region. As described in the section *Material and Methods*, we used Illumina reads of the same strain for this purpose. Because the focus of the present paper is the *Mst77Y* region (*i.e.*, the 96 kb located in coordinates 85040−180612 of contig JSAE01000257), we first analyzed it; the rest of the 747 kb contig will be dealt with in the next section.

**Figure 3:**
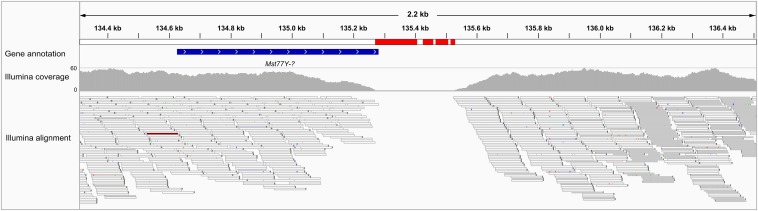
Misassemblies in a region of the PBcR assembly. This snapshot of the *IGV* browser shows a region of contig 0_176540 that seemed to contain a new *Mst77Y* gene (characterized by a 5′ deletion; labeled as “*Mst77Y-?*”). Note the “zero Illumina coverage” region and the presence of unmatched *k*-mers (marked in red), which show that the region was misassembled, and that the new gene is an artifact.

We found just two small errors in the *Mst77Y* region: a T insertion at 85,619 (in a run of five T) and C/T substitution at position 110,630, both in intergenic regions (Figure S1). Thus, the assembly of this region seems to be essentially perfect. This conclusion is strengthened by the analysis of the two preliminary PacBio assemblies (PBcR and FALCON), for we could detect many misassemblies there (Table 2 and Figure 3). In other words, the apparent absence of misassemblies in MHAP is not caused by a lack of power to detect them. Some misassemblies could have lead to wrong conclusions in biologically relevant issues: the first PacBio assembly we examined (PBcR) contain what seems to be a new *Mst77Y* gene, characterized by a deletion in the 5′ end; the Illumina reads shows that it was a misassembly (Figure 3). Given the data discussed here (Table 2) and in the previous section (Figure 2), we conclude that the MHAP assembly allows for at least a fairly accurate description of the *Mst77Y* region, and that it is unlikely that major amendments will be needed in the future.

**Table 2:** Assembly errors of PacBio assemblies in the *Mst77Y* region

Assembly	Contig	Coordinates	Unmatched *k*-mers	Regions With Zero Coverage	Total Base Pairs With Zero Coverage
MHAP	JSAE01000257	85040−180612	1	0	0
PBcR	0_176540	87315−173699	9	1	245
FALCON	0032_03	436429 −531977	36	11	138

### Content of the whole contig JSAE01000257

The MHAP assembly (and also FALCON) extends some 550 kb to the right of the *Mst77Y* region, allowing for the annotation and description of its surroundings. We found that part of this region has been previously sequenced and studied by Villasante *et al.*, and we direct the reader to their papers for detailed analyses (Agudo *et al.* 1999; Abad *et al.* 2004; Méndez-Lago *et al.* 2009). We will make here only a brief comparison of the shared sequences and point to the regions not sequenced in their study. Before doing this, it is important to note that there are clear signs of misassembly in PacBio assemblies of this broader region (Figure S1 and Table S1), so the results should be taken with a grain of salt. The main potential problem for the level of analysis we are doing in this broad region is not the collapsing of some repeat or some single-base error but rather a chimeric contig that would misjoin sequences that are physically very distant (*e.g.*, from other chromosome). We cannot exclude this possiblity of chimerism, given the misassembly signs and the lack of independent long-range data [*e.g.*, sequenced bacterial artificial chromosome (BACs)] for most of the length of contig JSAE01000257. On the other hand, the broad sequence composition we found in the whole contig JSAE01000257 is remarkably similar to preliminary data from a tiling path of BACs of this region (this section), which suggests that there are no major assembly errors.

Abad *et al.* (2004; see their Figure 1) presented a tiling path of BACs, presumably not sequenced, that would span the whole contig JSAE01000257. Méndez-Lago *et al.* (2009) sequenced twice one of these BACs (BACR26J21, from the ISO1 strain; accessions FM992409 and CU076040), which spans positions 280−445 kb of contig JSAE01000257. Figure S2 shows the dot plot alignment between contig JSAE01000257 and BACR26J21. The general agreement is very good, and no major misassembly such as chimeric regions was found in contig JSAE01000257. The largest discrepancy with BACR26J21 is in a region of contig JSAE01000257 (279−294 kb), which includes a zero Illumina coverage region, so the PacBio MHAP assembly almost certainly is wrong here (the alternative explanation would be mutation in the reference strain). As Méndez-Lago *et al.* (2009) described, this region is mostly composed of decayed telomeric transposons Het-A and TART, and the 18HT satellite, which derived from them.

Zooming out from the region covered by BACR26J21, we can recognize four broad “domains” in the whole sequence of contig JSAE01000257 (see also Figure 1 of Abad *et al.* 2004). The *Mst77Y* region detailed in the previous section extends from 85 to 181 kb. To its left (0−85 kb) and its immediate right (181−250 kb), and also between 559 kb and 685 kb, the sequence is almost entirely composed of transposons and a few pseudogenes, derived from the *Tequila*, *ade5*, *CG46192*, *CG12717*, and *Crg-1* genes. During previous searches of Y-linked genes we had found one of these pseudogenes (*ade5-ψ*, located at coordinates 17441−17643), and experimentally confirmed its Y-linkage (A. B. Carvalho, unpublished data). The third domain spans from 256 kb to 549 kb (and hence includes BACR26J21) and is almost entirely composed of sequences derived from telomeric transposons. The fourth domain starts at 685 kb and goes until the end of contig JSAE01000257 (at 747 kb); it is mostly composed of *rDNA* genes, with R1 and R2 insertions. The interesting possibility arises that contig JSAE01000257 captured the transition to the *rDNA* cluster of the Y chromosome [band h20; (Gatti and Pimpinelli 1983)], since the *Mst77Y* region has been mapped to h18-h19 bands (Russell and Kaiser 1993) and BACR26J21 to the h18 band.

One of the most interesting findings in this section is that the *Pp1-Y2* gene is located in contig JSAE01000257 (coordinates 565977−566918). This is a functional single-copy gene of the Y chromosome, which encodes a testis-specific protein phosphatase. It was described by Carvalho *et al.* (2001), who mapped it to the tip of the long arm of the Y (using the standard marked Y chromosome strains; Kennison 1981), whereas Abad, Villasante *et al.* have mapped it to h18 region (with BAC fluorescent *in situ* hybridization; Abad *et al.* 2004). Although the definitive experiment—fluorescent *in situ* hybridization using a *Pp1-Y2* probe—has yet to be performed, it seems reasonable to place this gene at h18; the results from Carvalho *et al.* (2001) would be explained by undetected rearrangements occurred during the obtainment of marked Y chromosome strains.

### Evolutionary analysis of the *Mst77Y* genes

Krsticevic *et al.* (2010; see their Figure 4) found four lines of evidence that strengthened the case that some *Mst77Y* are functional genes. Test 1 showed that as a whole (*i.e.*, combining potentially functional and non-functional ones) the *Mst77Y* genes evolved under purifying selection (*P* = 0.027); Test 2 showed that most (or all) purifying selection occurred in the potentially functional *Mst77Y* (*P* = 0.015); Test 3 found no evidence of purifying selection in non-functional *Mst77Y* (*P* = 0.54); and Test 4 showed that the potentially functional *Mst77Y* evolved under purifying selection (*P* = 0.017). Given that six of the 18 genes are artifacts (namely, *Mst77Y-2*, *Mst77Y-5*ψ, *Mst77Y-8*, *Mst77Y-9*, *Mst77Y-11*ψ, and *Mst77Y-14*ψ), it is desirable to repeat the aforementioned evolutionary analyses removing these six genes. When we did this (again using the HyPhy package; Pond *et al.* 2005), we found that Test 1 and Test 3 yield the same qualitative result (*P* = 0.046 and *P* = 0.246, respectively), but Test 2 and Test 4 are no longer statistically significant (*P* = 0.210 and *P* = 0.091, respectively). Table S2 detailed the updated tests; a comparison with Table S2 from Krsticevic *et al.* 2010 shows that the estimated parameters are similar. For example, the selective constraint ω of the potentially functional *Mst77Y* genes (ω = 0.54) still suggests stronger purifying selection compared with the nonfunctional ones (ω = 0.63), but the difference is no longer statistically significant. This pattern suggests that removal of the six spurious sequences reduced the statistical power of the tests. Indeed, simulations show that the power of Test 1 decreased from 89 to 39% and that a similar power reduction occurred with the other tests (File S1 and Table S4). Besides differences in statistical power, another factor that may explain the difference between the 2010 and the present study is that the six removed sequences are recombinants, and it is known that recombination interferes with likelihood methods for detecting selection pressure on codon alignments (Anisimova *et al.* 2003). It is worth to note that even after the removal of the six artifactual sequences there still is statistically significant evidence for recombination, as evidenced by the GARD method (*P* < 10^−3^; Pond *et al.* 2006). These recombination events, possibly due to gene conversion or transposable elements activity, may further reduce the power to detect purifying selection on the potentially functional *Mst77Y* genes.

We also analyzed our data using the recently published RELAX method, which has several advantages over the standard approaches for detecting relaxed selection (Wertheim *et al.* 2015). The results are shown in Table S3; although the face value of the “selection intensity” parameter *k* suggest that there is some selection acting on *Mst77Y* genes as a whole (*k* = 0.549, instead of 0, as expected for strict neutrality), and that the selection intensity is stronger in the potentially functional *Mst77Y* genes (*k* = 0.686), compared with the nonfunctional ones (*k* = 0.303), none of the effects is statistically significant.

We conclude from the aforementioned analyses that although there is some indication of purifying selection on *Mst77Y* genes, the evidence is not conclusive. Hence the results of the molecular evolutionary analysis are less compelling than those reported in Krsticevic *et al.* (2010). However, the main evidence that the “potentially functional” *Mst77Y* genes are functional genes is unchanged: they account for ∼20% of the *Mst77F*-like mRNA.

## Supplementary Material

Supporting Information
