# Supplementary material for: Long-Read Single Molecule Sequencing to Resolve Tandem Gene Copies: The Mst77Y Region on the Drosophila melanogaster Y Chromosome
Source: G3 (Bethesda). 2015 Apr 9;5(6):1145–50. doi: 10.1534/g3.115.017277 (PMC4478544; doi:10.1534/g3.115.017277)
Supplement: Supporting Information [file supp_5_6_1145__index.html]

Long-Read Single Molecule Sequencing to Resolve Tandem Gene Copies: The Mst77Y Region on the Drosophila melanogaster Y Chromosome — Supporting Information 

# Long-Read Single Molecule Sequencing to Resolve Tandem Gene Copies: The *Mst77Y* Region on the *Drosophila melanogaster* Y Chromosome

## Supporting Information for Krsticevic, Schrago, and Carvalho, 2015

**Files in this Data Supplement:**

- Supporting Information - Figures S1-S2, Tables S1-S4, and File S1 (PDF, 212 KB)
- Figure S1 - Assembly errors in contig JSAE01000257 (MHAP assembly). (PDF, 109 KB)
- Figure S2 - Comparison between contig JSAE01000257 (MHAP assembly) and BAC clone BACR26J21. (PDF, 95 KB)
- Table S1 - Assembly errors in the whole contig (FALCON/MHAP shared region). (PDF, 101 KB)
- Table S2 - Evolutionary analysis of the *Mst77Y* genes. (PDF, 137 KB)
- Table S3 - RELAX analysis of the *Mst77Y* genes. (PDF, 131 KB)
- Table S4 - Power and type I error of differential dN/dS tests. (PDF, 81 KB)
- File S1 - Supplementary Discussion (PDF, 157 KB)
