# Supplementary material for: Long-Read Single Molecule Sequencing to Resolve Tandem Gene Copies: The Mst77Y Region on the Drosophila melanogaster Y Chromosome
Source: G3 (Bethesda). 2015 Apr 9;5(6):1145–50. doi: 10.1534/g3.115.017277 (PMC4478544; doi:10.1534/g3.115.017277)
Supplement: Supporting Information [file supp_g3.115.017277_017277SI.pdf]

G3 · Genes | Genomes | Genetics

Long-read single molecule sequencing to resolve tandem gene copies: The *Mst77Y* region on the *Drosophila melanogaster* Y chromosome

Flavia J. Krsticevic[*]*, Carlos G. Schrago*[§] *and A. Bernardo Carvalho*[§,1]

* Centro Internacional Franco Argentino de Ciencias de la Información y de Sistemas - CONICET. Ocampo y Esmeralda, S2000EZP Rosario, Argentina.

§ Departamento de Genética, Universidade Federal do Rio de Janeiro, Caixa Postal 68011 CEP 21941-971, Rio de Janeiro, Brazil.

[1] Corresponding author. E-mail: bernardo@biologia.ufrj.br ; bernardo1963@gmail.com

DOI: 10.1534/g3.115.017277

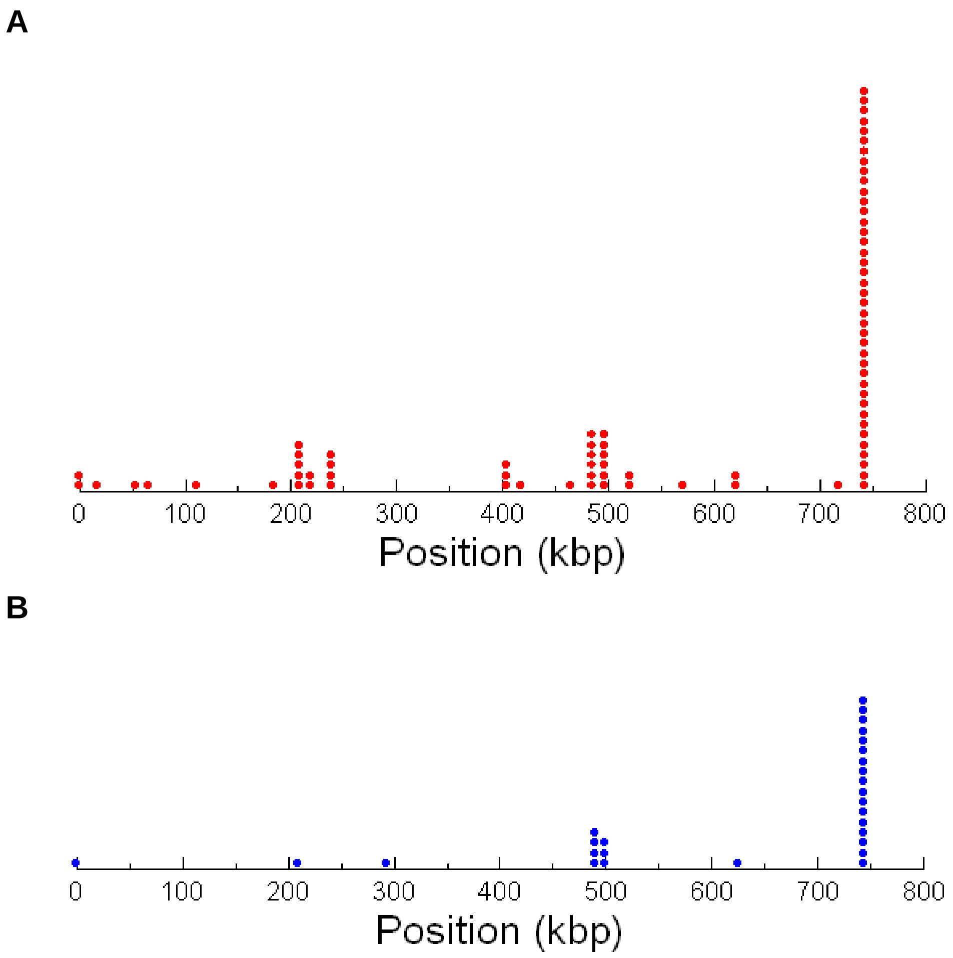

Figure S1 Assembly errors in contig JSAE01000257 (MHAP assembly). Assembly errors were identified by comparison with Illumina short reads using two methods. (A) Unmatched *k*-mers (*YGS* program; see Material and Methods). (B) Runs of zero Illumina coverage (*bwa* alignment). The *Mst77Y* region, which spans from 85kb to 181kb, has very few errors: the two methods detected only one error (an unmatched *k*-mer caused by a C/T substitution at position 110,630) , whereas detailed inspection with the *IGV* browser revealed a T insertion at 85,619 (in a run of five T). The two errors are in intergenic regions; note that both could actually be residual polymorphisms or new mutations in the sequenced strain. Contig size is 747 kb.

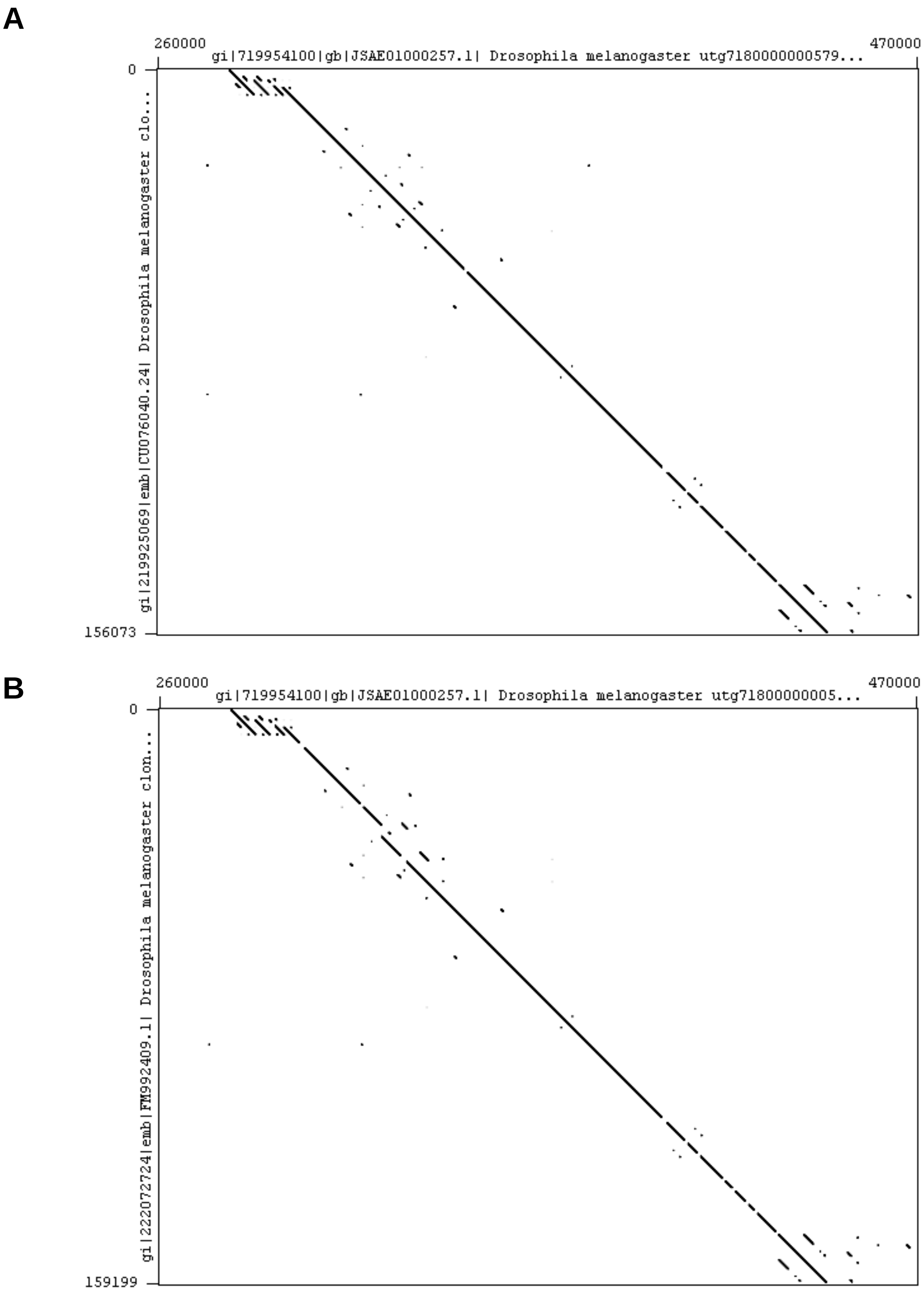

Figure S2 Comparison between contig JSAE01000257 (MHAP assembly) and BAC clone BACR26J21. The BAC clone BACR26J21 was sequenced twice, with a few discrepancies between the two versions (Mendez-Lagos et al 2009). (A) BAC assembly CU076040 (B) BAC assembly FM992409. Note the absence of gross assembly errors in contig JSAE01000257, such as chimeric regions. The largest discrepancy lies around position 292 kb of contig 579 and likely resulted from collapsing some copies of the 18HT satellite. There is a zero coverage stretch in this region (between positions 291878-291919 of contig 579), so the PacBio assembly probably is wrong here. Note also that this discrepancy could actually be residual polymorphisms or new mutations in the sequenced strain. Dot plot done with word size set to 1000 to remove the cluttering due to repetitive regions.

TABLE S1 Assembly errors in the whole contig (FALCON/MHAP shared region).

Assembly	Contig	Coordinates	Unmatched *k*-mers	Regions with zero coverage	Total bp with zero coverage
MHAP	JSAE01000257	1 - 713441	40	11	239
FALCON	0032_03	1 - 616983	288	59	1330

TABLE S2 Evolutionary analysis of the *Mst77Y* genes.

Test	Hypothesis	Parameter constraints				Parameter values						χ^2 (*d.f.*)	*P*-value
		dN_{pf}	dS_{pf}	dN_{nf}	dS_{nf}	dN_{pf}	dS_{pf}	dN_{nf}	dS_{nf}	ω_{pf}	ω_{nf}		
1	H_0	free	$= dN_{pf}$	$= dN_{pf}$	$= dN_{pf}$	10.46	10.46	10.46	10.46	1	1	3.99 (1)	0.046
	H_1	free	free	$= dN_{pf}$	$= dS_{pf}$	9.06	15.30	9.06	15.30	0.59	0.59		
2	H_0	free	free	$= dN_{pf}$	$= dS_{pf}$	9.06	15.30	9.06	15.30	0.59	0.59	3.12 (2)	0.210
	H_1	free	free	free	free	7.29	13.49	11.68	18.39	0.54	0.63		
3	H_0	free	free	free	$= dN_{nf}$	7.30	13.70	13.03	13.03	0.53	1	1.34 (1)	0.246
	H_1	free	free	free	free	7.29	13.49	11.68	18.39	0.54	0.63		
4	H_0	free	$= dN_{pf}$	free	$= dN_{nf}$	8.66	8.66	13.14	13.14	1	1	2.85 (1)	0.091
	H_1	free	free	free	$= dN_{nf}$	7.30	13.70	13.03	13.03	0.53	1		

TABLE S3 RELAX analysis of the *Mst77Y* genes.

Reference	Test	Unclassified	*k*	*P*
other branches	Ypf + Ynf	-	0.549	0.245
Ypf	Ynf	other branches	0.397	0.445
other branches	Ynf	Ypf	0.303	0.258
other branches	Ypf	Ynf	0.686	0.503

The RELAX method (Wherteim et al. 2014) compares Reference (background) branches with Test branches; optionally some branches of the phylogeny (labeled as "Unclassified") may be excluded from the analysis. The selection intensity parameter k measures the relaxation of selection (both purifying and positive); relaxed selection appears as $k < 1$, strict neutrality as $k = 0$, and intensified selection as $k > 1$. The null hypothesis is $k = 1$.

TABLE S4 Power and type I error of differential dN/dS tests.

Test	Dataset	Type I error	Power
1	Large	0.06	0.89
	Small	0.06	0.39
2	Large	0.08	0.68
	Small	0.07	0.34
3	Large	0.07	0.39
	Small	0.02	0.02
4	Large	0.04	0.85
	Small	0.16	0.45

File S1
Supplementary Discussion

Estimation of power and error frequency of differential d_N/d_S tests:

To estimate the power and error frequencies of the four tests described in Figure 4 of Krsticevic et al. (2010), we simulated sequences in HyPhy 2.2 (Pond *et al.* 2005) under the models proposed by the authors. In all simulations, we adopted parametric estimates of d_N and d_S as inferred from the empirical data. The MG94xHKY85_3x4 model of codon substitution was used. Simulations were conducted with both the alignment including *Mst77Y* sequences from Krsticevic et al. (2010) and the alignment of sequences reported in the present study. These datasets were dubbed large and small respectively. Estimation of the power and error rates of differential selection tests were based on 100 simulated replicates.

The frequency of false positives (Type I error) was estimated by the frequency of replicates that rejected the null hypothesis in each test, when sequences were simulated under that same null model. Type II error of tests was estimated by the frequency of replicates that failed to reject the null model, when sequences were simulated under the alternative hypothesis. Power of tests was calculated from the inferred Type II errors ($1 - \beta$). Table S4 shows the results. Note the strong reduction is statistical power in the small dataset (12 *Mst77Y* sequences) when compared to the large dataset (18 *Mst77Y* sequences).
