# Supplementary material for: Long-Read Single Molecule Sequencing to Resolve Tandem Gene Copies: The Mst77Y Region on the Drosophila melanogaster Y Chromosome
Source: G3 (Bethesda). 2015 Apr 9;5(6):1145–50. doi: 10.1534/g3.115.017277 (PMC4478544; doi:10.1534/g3.115.017277)
Supplement: Supporting Information [file supp_g3.115.017277_FigureS1.pdf]

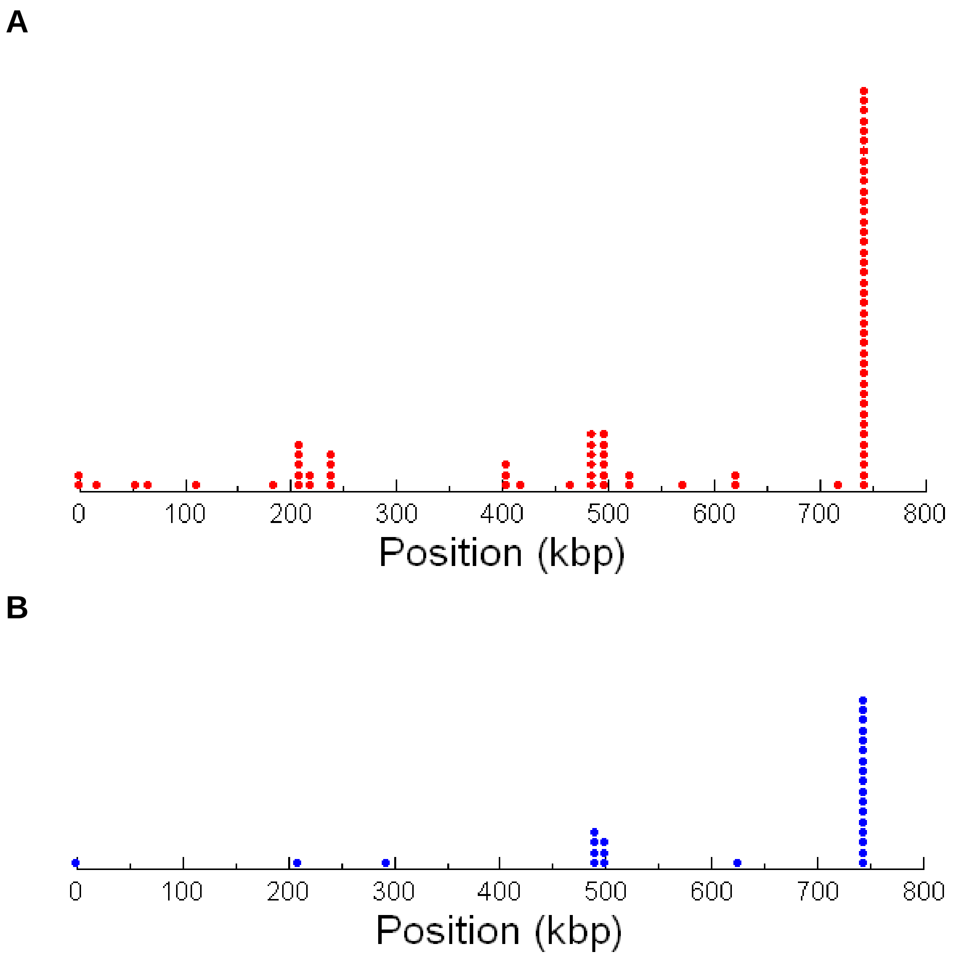

Figure S1 Assembly errors in contig JSAE01000257 (MHAP assembly). Assembly errors were identified by comparison with Illumina short reads using two methods. (A) Unmatched *k*-mers (*YGS* program; see Material and Methods). (B) Runs of zero Illumina coverage (*bwa* alignment). The *Mst77Y* region, which spans from 85kb to 181kb, has very few errors: the two methods detected only one error (an unmatched *k*-mer caused by a C/T substitution at position 110,630) , whereas detailed inspection with the *IGV* browser revealed a T insertion at 85,619 (in a run of five T). The two errors are in intergenic regions; note that both could actually be residual polymorphisms or new mutations in the sequenced strain. Contig size is 747 kb.
