# Supplementary material for: Long-Read Single Molecule Sequencing to Resolve Tandem Gene Copies: The Mst77Y Region on the Drosophila melanogaster Y Chromosome
Source: G3 (Bethesda). 2015 Apr 9;5(6):1145–50. doi: 10.1534/g3.115.017277 (PMC4478544; doi:10.1534/g3.115.017277)
Supplement: Supporting Information [file supp_g3.115.017277_FigureS2.pdf]

A

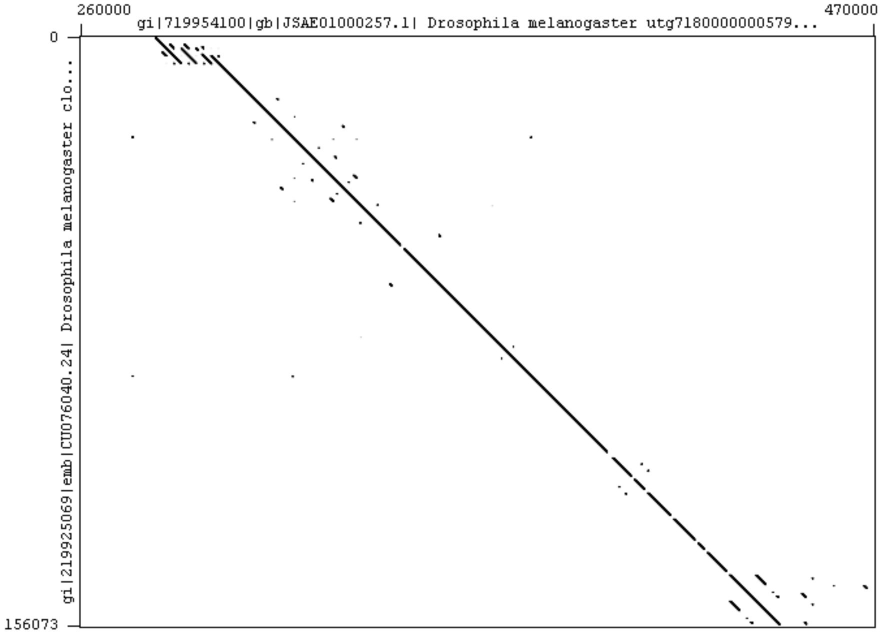

B

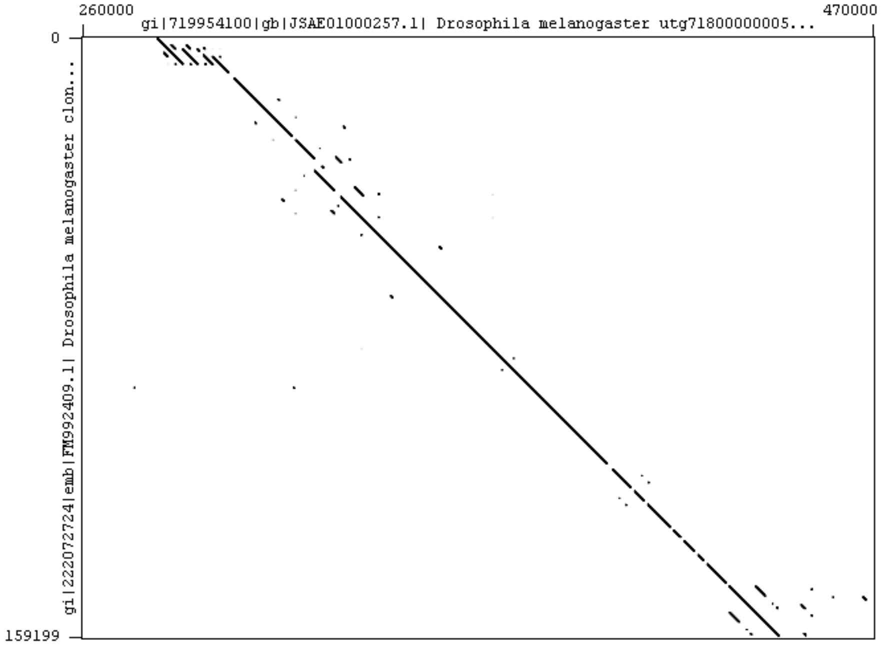

Figure S2 Comparison between contig JSAE01000257 (MHAP assembly) and BAC clone BACR26J21. The BAC clone BACR26J21 was sequenced twice, with a few discrepancies between the two versions (Mendez-Lagos et al 2009). (A) BAC assembly CU076040 (B) BAC assembly FM992409. Note the absence of gross assembly errors in contig JSAE01000257, such as chimeric regions. The largest discrepancy lies around position 292 kb of contig 579 and likely resulted from collapsing some copies of the 18HT satellite. There is a zero coverage stretch in this region (between positions 291878-291919 of contig 579), so the PacBio assembly probably is wrong here. Note also that this discrepancy could actually be residual polymorphisms or new mutations in the sequenced strain. Dot plot done with word size set to 1000 to remove the cluttering due to repetitive regions.
