# Supplementary material for: Long-Read Single Molecule Sequencing to Resolve Tandem Gene Copies: The Mst77Y Region on the Drosophila melanogaster Y Chromosome
Source: G3 (Bethesda). 2015 Apr 9;5(6):1145–50. doi: 10.1534/g3.115.017277 (PMC4478544; doi:10.1534/g3.115.017277)
Supplement: Supporting Information [file supp_g3.115.017277_TableS1.pdf]

TABLE S1 Assembly errors in the whole contig (FALCON/MHAP shared region).

Assembly	Contig	Coordinates	Unmatched *k*-mers	Regions with zero coverage	Total bp with zero coverage
MHAP	JSAE01000257	1 - 713441	40	11	239
FALCON	0032_03	1 - 616983	288	59	1330
