# Supplementary material for: Long-Read Single Molecule Sequencing to Resolve Tandem Gene Copies: The Mst77Y Region on the Drosophila melanogaster Y Chromosome
Source: G3 (Bethesda). 2015 Apr 9;5(6):1145–50. doi: 10.1534/g3.115.017277 (PMC4478544; doi:10.1534/g3.115.017277)
Supplement: Supporting Information [file supp_g3.115.017277_TableS2.pdf]

TABLE S2 Evolutionary analysis of the *Mst77Y* genes.

Test	Hypothesis	Parameter constraints				Parameter values						χ^2 (*d.f.*)	*P*-value
		dN_{pf}	dS_{pf}	dN_{nf}	dS_{nf}	dN_{pf}	dS_{pf}	dN_{nf}	dS_{nf}	ω_{pf}	ω_{nf}		
1	H_0	free	$= dN_{pf}$	$= dN_{pf}$	$= dN_{pf}$	10.46	10.46	10.46	10.46	1	1	3.99 (1)	0.046
	H_1	free	free	$= dN_{pf}$	$= dS_{pf}$	9.06	15.30	9.06	15.30	0.59	0.59		
2	H_0	free	free	$= dN_{pf}$	$= dS_{pf}$	9.06	15.30	9.06	15.30	0.59	0.59	3.12 (2)	0.210
	H_1	free	free	free	free	7.29	13.49	11.68	18.39	0.54	0.63		
3	H_0	free	free	free	$= dN_{nf}$	7.30	13.70	13.03	13.03	0.53	1	1.34 (1)	0.246
	H_1	free	free	free	free	7.29	13.49	11.68	18.39	0.54	0.63		
4	H_0	free	$= dN_{pf}$	free	$= dN_{nf}$	8.66	8.66	13.14	13.14	1	1	2.85 (1)	0.091
	H_1	free	free	free	$= dN_{nf}$	7.30	13.70	13.03	13.03	0.53	1		
