# Supplementary material for: Long-Read Single Molecule Sequencing to Resolve Tandem Gene Copies: The Mst77Y Region on the Drosophila melanogaster Y Chromosome
Source: G3 (Bethesda). 2015 Apr 9;5(6):1145–50. doi: 10.1534/g3.115.017277 (PMC4478544; doi:10.1534/g3.115.017277)
Supplement: Supporting Information [file supp_g3.115.017277_TableS3.pdf]

TABLE S3 RELAX analysis of the *Mst77Y* genes.

Reference	Test	Unclassified	*k*	*P*
other branches	Ypf + Ynf	-	0.549	0.245
Ypf	Ynf	other branches	0.397	0.445
other branches	Ynf	Ypf	0.303	0.258
other branches	Ypf	Ynf	0.686	0.503

The RELAX method (Wherteim et al. 2014) compares Reference (background) branches with Test branches; optionally some branches of the phylogeny (labeled as "Unclassified") may be excluded from the analysis. The selection intensity parameter k measures the relaxation of selection (both purifying and positive); relaxed selection appears as $k < 1$, strict neutrality as $k = 0$, and intensified selection as $k > 1$. The null hypothesis is $k = 1$.
