# Supplementary material for: Long-Read Single Molecule Sequencing to Resolve Tandem Gene Copies: The Mst77Y Region on the Drosophila melanogaster Y Chromosome
Source: G3 (Bethesda). 2015 Apr 9;5(6):1145–50. doi: 10.1534/g3.115.017277 (PMC4478544; doi:10.1534/g3.115.017277)
Supplement: Supporting Information [file supp_g3.115.017277_TableS4.pdf]

TABLE S4 Power and type I error of differential dN/dS tests.

Test	Dataset	Type I error	Power
1	Large	0.06	0.89
	Small	0.06	0.39
2	Large	0.08	0.68
	Small	0.07	0.34
3	Large	0.07	0.39
	Small	0.02	0.02
4	Large	0.04	0.85
	Small	0.16	0.45
