# Supplementary material for: Long-Read Single Molecule Sequencing to Resolve Tandem Gene Copies: The Mst77Y Region on the Drosophila melanogaster Y Chromosome
Source: G3 (Bethesda). 2015 Apr 9;5(6):1145–50. doi: 10.1534/g3.115.017277 (PMC4478544; doi:10.1534/g3.115.017277)
Supplement: Supporting Information [file supp_g3.115.017277_FileS1.pdf]

File S1
Supplementary Discussion

Estimation of power and error frequency of differential d_N/d_S tests:

To estimate the power and error frequencies of the four tests described in Figure 4 of Krsticevic et al. (2010), we simulated sequences in HyPhy 2.2 (Pond *et al.* 2005) under the models proposed by the authors. In all simulations, we adopted parametric estimates of d_N and d_S as inferred from the empirical data. The MG94xHKY85_3x4 model of codon substitution was used. Simulations were conducted with both the alignment including *Mst77Y* sequences from Krsticevic et al. (2010) and the alignment of sequences reported in the present study. These datasets were dubbed large and small respectively. Estimation of the power and error rates of differential selection tests were based on 100 simulated replicates.

The frequency of false positives (Type I error) was estimated by the frequency of replicates that rejected the null hypothesis in each test, when sequences were simulated under that same null model. Type II error of tests was estimated by the frequency of replicates that failed to reject the null model, when sequences were simulated under the alternative hypothesis. Power of tests was calculated from the inferred Type II errors ($1 - \beta$). Table S4 shows the results. Note the strong reduction is statistical power in the small dataset (12 *Mst77Y* sequences) when compared to the large dataset (18 *Mst77Y* sequences).
